# Accessibility of healthcare services for seasonal migrant farmworkers in Spain: barriers and facilitators identified by professionals

**DOI:** 10.1016/j.jmh.2025.100353

**Published:** 2025-08-18

**Authors:** Lena van Selm, Iratxe Pérez-Urdiales, José Tomás Mateos, Maria del Mar Jiménez-Lasserrotte, María del Mar Pastor-Bravo, Ana Requena-Méndez, Erica Briones-Vozmediano

**Affiliations:** a Barcelona Institute for Global Health (ISGlobal), Barcelona, Spain; b Department of Nursing I, University of the Basque Country (UPV/EHU), Biscay, Spain; c Biocruces Bizkaia Health Research Institute, Biscay, Spain; d Department and faculty of nursing and physiotherapy. Consolidated Research Group in Society, Health, Education and Culture (GESEC), University of Lleida, Lleida, Spain; e Research Group in Healthcare (GRECS), Biomedical Research Institute (IRB) Lleida, Spain; f Department of Nursing, Physiotherapy, and Medicine, University of Almería, Almería, Spain; g Department of Nursing, University of Murcia, Cartagena (Murcia), Spain; h Department of Medicine Solna, Karolinska Institute, Stockholm, Sweden; i Biomedical Research Networking Center of Infectious Diseases, (CIBERINFEC), Barcelona, Spain

**Keywords:** Transients and migrants, Agricultural workers' diseases, Farmers, Health services accessibility, Qualitative Research

## Abstract

**Introduction:**

In Europe, Spain has the highest percentage of migrants working in agriculture. Seasonal migrant farmworkers (SMFs) are a vulnerable group in the labor market as they often work in precarious situations that may impact their health. Although all migrants with municipality registrations, including those in irregular situations, should have access to healthcare according to Spanish law, some barriers limit access to care for migrants. This study aimed to identify barriers and facilitators to SMFs' accessibility to healthcare services at the healthcare system level.

**Methods:**

We conducted semi-structured interviews among a purposive sample of 92 professionals from the health and social support system who work with this population in four regions of Spain. Using Atlas.ti web, we analyzed the transcriptions using a thematic content analysis approach.

**Results:**

The main barriers identified were administrative, geographical, time, financial, and healthcare system capacity. Subsequently, SMFs may use emergency care to enter the healthcare system and, for non-urgent issues, some of them were using other people´s documents when using healthcare facilities. Facilitators identified were reducing administrative barriers, providing patient-centered care, and supporting services provided by NGOs.

**Conclusion:**

To increase access to healthcare services, more flexibility is required regarding issuing municipality registrations and healthcare cards and appointment schedules. Some of the administrative barriers identified in this study are rooted in municipal authorities not consistently complying with recommendations and regulations by the Spanish government. Therefore, consistent compliance by public officials throughout all regions would be the first step in increasing access to healthcare.

## Highlights

• Seasonal Migrant Farmworkers (SMF) face barriers accessing healthcare services.
• Barriers to healthcare are administrative, geographical, time and financial.
• To overcome barriers, SMFs often access healthcare systems through emergency care.
• Collaboration between healthcare centers and NGOs facilitate SMFs' healthcare access.
• Spanish regions should improve collaboration and simplify administrative processes.

## 1 Introduction

Europe has a long history of migration. In 2022, among the inhabitants living in the European Union (EU), 8.5 % of the population was born outside the EU (European Commission 2023). Migrants often work in temporary, informal, or unprotected jobs, exposing them to a high risk of social insecurity and layoffs (International Labour Organization 2021). In the EU, migrants are overrepresented in elementary, physically demanding sectors such as cleaning, personal services and care, construction, and agriculture (European Commission 2023).

In Spain, according to official numbers, 24.2 % of agricultural workers are migrants, representing 188,900 people (Instiuto Nacional de Estadística 2024). However, this is an underestimation since undocumented migrant workers performing informal labour are not included in these numbers. In 2019, it was estimated that 37.8 % of the agriculture sector in Spain consisted of informal labor (Williams, 2019).

The working conditions of seasonal migrant farmworkers (SMFs) in Spain depend on the type of contract they are eligible for (Hooper and Le Coz, 2020). Intra-EU workers have the same rights as local citizens (European Parliament 2021). Some people from non-EU countries, such as Morocco, Colombia, and Ecuador, are recruited in the countries of origin through circular recruitment (Organisation for Economic Co-operation and Development (OECD) 2022). Furthermore, many SMFs have an irregular administrative status, meaning their labor rights and access to health and social assistance are not regulated.

SMF characteristics, origins and their working situations differ between regions in Spain. In Andalusia there is a high percentage of Moroccan women working with red fruits in Huelva and men from Morocco and sub-Saharan Africa working year-round in greenhouses in Almería. (Briones-Vozmediano and González-González, 2022; Plaza Del Pino et al., 2023) Workers are almost exclusively migrants. Approximately 7000 in Almería and 5000 in Huelva live in informal shanty towns close to the agricultural sites (Pedreño Cánovas, 2020). In Lleida, Catalonia, work is mainly seasonal, with an estimated 20,000 people working during the harvesting season in summer, mostly male workers from sub-Saharan Africa (Briones-Vozmediano and González-González, 2022; González Rodríguez et al., 2021). Many undocumented migrant workers move between regions for temporary jobs during the different harvesting seasons (Gadea et al., 2015).

Several studies in Spain showed that migrants often live in precarious situations, living in overpopulated housing in remote areas, having low salaries, and without rights to benefits when they lose their jobs (Briones-Vozmediano and González-González, 2022; Briones-Vozmediano et al., 2022). Several factors, such as irregular migrant status, low education levels, and high economic needs, force migrants to accept low-paid jobs under unfavorable conditions that negatively impact their health (HN Urrego-Parra et al., 2022). Also, limited control over workplace conditions and language and cultural barriers increases the risk of occupational injuries and diseases (El Khayat et al., 2022). Among agricultural workers, migrants had 91 % more accidents than Spanish natives between 2013–2018 (Baraza and Cugueró-Escofet, 2022). Therefore, SMFs are at increased risk of adverse health outcomes, and access to healthcare for this population is essential.

In Spain, the National Health System is decentralized, and each of the 17 autonomous regions has its own public health system. According to the state 2018 Royal Decree-Law, everyone in Spain can access healthcare services. However, except for emergency care, pregnancy care, and healthcare for minors, a healthcare card is required to obtain care, for which proof of residence in Spain for at least three months is required. Therefore, although theoretically healthcare is available to all migrants, including those with an irregular status, some requirements limit their access to practice (Hsia and Gil-González, 2021).

In this sub-study of the broader AGROMISALUD project (Agricultural work and migration in Spain: socio-labor precariousness and health) (Briones-Vozmediano et al., 2022), we explored barriers and facilitators for SMFs to access healthcare services in Spain from the perspective of professionals who work with this population in four autonomous communities in Spain.

## 2 Methods

In this descriptive qualitative study, data was collected between January and October 2021 in four Spanish autonomous regions: two in the south; Andalusia (Huelva and Almería) and Murcia, and two in the north; Catalonia (Lleida) and La Rioja. These areas comprised the highest concentration of SMFs and agricultural sites in Spain. An intentional sampling strategy was applied, selecting professionals with at least one year of experience working with SMFs, and snowball recruitment technique was used. To capture a wide range of views, both professionals working directly with SMF (e.g., NGO employees and healthcare workers) and professionals involved in policy making and execution (e.g., labor unions and politicians) were included.

### 2.1 Data collection

The researchers contacted the professionals directly by email or phone. First, the aim of the interview and of the study was explained, data confidentiality was guaranteed, and written informed consent was signed by participants. All interviews were performed online due to the COVID-19 pandemic measures and had a duration of between 45 and 90 min. A script of open-ended questions was used based on 5 thematic blocks: working with SMFs, migratory journey, living conditions, health and access to health services, and needs for improvement. Thematic saturation was achieved when no new themes, patterns, or insights emerged from additional data analysis, indicating that the data sufficiently captured the range of perspectives relevant to the study objectives.

### 2.2 Data analysis

We transcribed the interviews verbatim in Spanish and imported them into ATLAS.ti web. We conducted a thematic content analysis following Braun & Clarke (Braun and Clarke, 2021). First, we generated a coding tree based on the script to identify the main ideas in a preliminary analysis of the transcriptions. Then, we created additional open codes summarizing the content of the sentences. Two authors agreed on subthemes and associated them with possible themes. Then, three additional researchers independently verified the transcripts, the generated subthemes and themes, and the coherence of the related citations. The team refined the initial themes and subthemes and validated the final ones.

### 2.3 Rigor

Methodological rigor was ensured throughout the study following the Guba and Lincoln quality criteria (Lincoln and Guba, 1985). Credibility was enhanced by the triangulation of the interpretation of the analysis results among different researchers and by providing verbatim quotes in the results section to support their joint interpretation. Dependability was guaranteed as five authors collected and codified the data independently. Then, two authors made independent proposals for the results, and all authors discussed and agreed upon the most appropriate one. Confirmability was enhanced as the authors have lengthy experience in qualitative and migration research and by the heterogeneity of the participant profiles. Transferability was achieved through the context description and the participant profiles. We followed the Consolidated Criteria for Reporting Qualitative Research (COREQ) to conduct and report the study (Tong et al., 2007).

### 2.4 Patient and public involvement

Research questions and interview script were developed by the research team. The study first involved the public when the script was piloted, so participants were asked if they missed any relevant issue that had not been treated during the interview. Moreover, as snowball recruitment technique was used, participants had the opportunity to suggest the inclusion of new potential participants.

### 2.5 Ethics approval

This study was performed in line with the principles of the Declaration of Helsinki. Approval was granted by the Healthcare Ethics Committee of the Arnau of Vilanova Hospital in Lleida in 2021 [CEIC-2459].

## 3 Results

Ninety-five professionals were invited to participate in the interviews, of which 92 participated (80 individual interviews and 6 interviews with 2 participants (Table 1). Three could not participate due to work commitments. Supplementary figure 1 shows the number of mentions per sub-theme.

**Table 1:** Sociodemographic characteristics of the study participants (*n* = 92).

	Autonomous region				
	Andalusia	Catalonia	La Rioja	Murcia	Total
Sex					
*Female*	25	10	13	7	55
*Male*	6	14	7	10	37
Sector of employment					
*Healthcare*	9	9	3	2	23
*Public Social services*	1	0	2	0	3
*NGOs*	21	11	5	6	43
*Worker Unions*	0	2	6	9	17
*Governmental institutions*	0	3	2	1	6

We structured the results under three themes, and nine emerging sub-themes related to barriers and facilitators for accessing healthcare services (Table 2).

**Table 2:** Themes, subthemes, categories and representative quotes.

THEME	SUBTHEME	CATEGORY
Barriers for accessing healthcare services	Administrative barriers: The right documentation to access	Obtaining a healthcare card
		Obtaining a municipality registration (empadronamiento)
		Moving between autonomous communities
		Difficulties to access healthcare after occupational accidents and illnesses
	Geographical and time barriers: When and how to access health services	Transport
		Opening hours of healthcare services
	Financial barriers	
	Healthcare system capacity/staff	
Strategies	Entering the system through emergency care	
	Entering healthcare with other people´s documents.	
Facilitators and improvement suggestions for accessing healthcare services	Reducing administrative barriers to healthcare	Reinforcing universal access
		Simplifying process of obtaining healthcare cards
		Permanent health card after obtaining permanent residence
	Patient centered care	Willingness of healthcare service personnel to attend migrants
		Attendance without appointments
	Support services by NGOs	Support obtaining healthcare cards
		Support with healthcare visits
		Logistical and financial support
		Collaboration between healthcare centers and NGOs
	Professionals’ suggestions for improvement of healthcare for SMFs	

### 3.1 Barriers for accessing healthcare services

#### 3.1.1 Administrative barriers

##### 3.1.1.1 Obtaining a healthcare card

A healthcare card entitles individuals to access healthcare services through the National Health System. The process of obtaining the healthcare card varied between autonomous regions. In the provinces included in our study, except for Andalusia, proof of registration in a municipality of the region was required to obtain a healthcare card.

“[To be attended to in the health system] *The first thing is to have a healthcare card, but to get one you need to have a municipality registration*.” – E32, Cultural mediator, Lleida

Despite this not being a legal requirement in Andalusia, several informants said that in certain centers, municipality registration was still being requested, for example, in centers overwhelmed with high numbers of migrants or that have had problems with migrants using other people’s healthcare card. In some other regions, the requirement to present the municipality registration had become stricter. Participants from Murcia explained that while it used to be possible to get a healthcare card without a municipality registration, it was now impossible. Likewise, participants from La Rioja explained that there used to be a special regulation for temporary workers that allowed them to access healthcare services without providing a municipality registration, but it was abolished.

##### 3.1.1.2 Obtaining a municipality registration

According to the participants, getting a municipality registration was difficult for SMFs. SMFs often rented housing without a formal contract and landlords usually did not allow them to register. The issue of local people not wanting to rent rooms to migrants was also mentioned. Subsequently, many SMFs were forced to live in informal housing, where municipality registration was not allowed, including shanty towns (more common in Andalusia and Murcia), garages and abandoned houses. In La Rioja and Catalonia, farmers were obliged by law to provide housing to workers, but only during the working period, which does not allow them to register there.

“ … *because they often go to live in houses that are not houses. In garages, pig farms… so they can’t [register]*.” – E21, Social worker, Murcia

Another identified barrier for SMFs was requirement of a valid identity document in order to register. Finally, moving throughout the country following the different harvesting seasons made it difficult to register anywhere.

“*Seasonal workers are constantly on the move… so where do they register*?” – E22, NGO, La Rioja

##### 3.1.1.3 Moving between autonomous regions

Each autonomous region had its own healthcare cards; therefore, difficulties arose when SMFs moved between autonomous regions. When possessing a healthcare card and moving to another autonomous region, a displacement healthcare card needed to be requested which is a complicated procedure for someone unfamiliar with the system.

“*Even if you have your healthcare card [from another autonomous region], If you do not have a general practitioner in Murcia, you cannot access a GP nor a specialist… you can only get emergency care.*” – E75, social worker, Murcia

A participant explained that in Murcia, Spanish-born people could usually get a displaced person’s card without being asked for additional documentation, while migrants would rarely get one without showing a municipality registration. In contrast, in La Rioja, migrants could get a displaced healthcare card when showing a healthcare card from a different autonomous community; however, many SMFs, especially those who had recently arrived, were unaware of this.

Participants mentioned that moving between regions complicated follow-up care for SMFs. Healthcare services could not access patients’ clinical histories from other autonomous regions without having the patient request this data, for which an identity document was required. These requests take time to process so the information was not instantly available. A healthcare worker in La Rioja said no national system to monitor between autonomous communities was available.

“*In our case, there is no official coordination [between regional health systems], unless it is a communicable disease for which we have to activate a protocol or do a follow-up treatment.*” – E84, healthcare professional, La Rioja

##### 3.1.1.4 Difficulties to access healthcare after occupational accidents and illnesses

In Spain, workers who suffer occupational accidents or diseases should be provided a doctor from the mutual insurance company. However, irregular workers cannot access labor insurance companies to get coverage for the expenses of their occupational injuries and diseases. Examples from participants showed some employers' negligence in facilitating access to healthcare for SMFs with no working contract, which could even lead to death.

“*Yes, in [name of the town], a worker died; it seems that he was not registered with social security, and they left him abandoned at the door of a [healthcare] center and he died /…/ if a worker is not registered and falls or has an accident, imagine what the employer will say, "I don't know him".*” E77, member agricultural labour union, Murcia

An agricultural labor union member mentioned getting many complaints from SMFs who were not well attended to and got told by the *labor insurance company* that their problems were not severe and that they could work, denying them their sick leave.

#### 3.1.2 Geographical and time barriers: when and how to access health services

##### 3.1.2.1 Transport

Many SMFs lived in shanty towns or places close to work, often with no healthcare facilities nearby. Participants said that companies would sometimes bring SMFs to the health centers. Some participants described how companies only responded when they considered the health problems to be serious and sometimes underestimated workers' health concerns.

“*We have seen that the agricultural employers have only reacted when the illnesses were severe, when the risk has been, as I said, almost of life or death. Apart from this, there is the macho attitude that we have heard: that they [women] are always complaining, they are always saying: "Oh, this hurts me, that hurts me…".”* - E29, NGO, Huelva

As a result, people were often forced to pay for transport to reach healthcare facilities, which could cause a financial barrier as an informal taxi would cost 5 to 10 euros each way. One midwife also explained that many migrant women have trouble reaching the hospital because they depend on their husbands to drive them.

##### 3.1.2.2 Opening hours of healthcare services

Since SMFs were working long days, far away from the healthcare centers, they could often not attend primary or specialist care during regular opening hours.

“*They leave in the morning and come back in the evening tired, they often leave their ailments for another day, another day, until one day, a problem comes to light that could have been stopped earlier.*” E73, NGO, Murcia

#### 3.1.3 Financial barriers

In Spain emergency care can be accessed for free even without a healthcare card. However, SMF would have to pay the prescribed medication, which could be even 100 % more than in the public system. With a healthcare card, the costs for most medications are partially funded by the public system. Although the costs in these cases were minimal, professionals mentioned examples of SMFs that stopped taking medicines because of the costs.

“*It is more difficult to control this hypertension; I mean, they stop taking the drugs because, even though they already have a minimal cost, they still have a cost, so they stop; it is not a priority for their life.*” - E5, healthcare worker, Lleida

#### 3.1.4 Healthcare system capacity/staff

Healthcare workers often had a high workload and limited capacity to provide more extensive explanations to migrants with limited understanding of the healthcare system or language. A healthcare worker explained that the SMF population heavily increased during the harvest season in some towns in Catalonia while the healthcare center capacity remained the same. This would lead to many centers being overburdened, mainly the emergency department, which is how most migrants entered the system, especially at the end of the day when many MSFs with occupational injuries come in following their shifts.

“*The emergency rooms fill up in the afternoon. People with pains, especially work-related pains, and infections… Things like that*.” E49, social worker, Andalusia

As many SMFs are not in the system, administrative procedures take longer, increasing the burden on administrative personnel at healthcare centers. NGO employees mentioned that although doctors and nurses are usually willing to attend migrant patients, the administrative personnel can be less collaborative. Examples cited included the lack of flexibility in dealing with language barriers and strict documentation requirements since bringing the proper forms can be challenging for migrants.

### 3.2 Strategies By SMFs To overcome access barriers

#### 3.2.1 Entering the system through emergency care

SMFs often got to the emergency care unit for work-related complaints more suitable for primary care, like lower back or hand and wrist pain. The participants explained that there were two main reasons for this: not understanding the Spanish system or being unable to attend primary care, either because they did not have a healthcare card or did not want to miss work.

*“They don't know how the system works, and they go directly through the emergency department /…/ it is explained to them how it works and it turns out that it is not that they don't want to go to the family doctor or that they think that the emergency department is quicker, but that they don't know that they have that option…”* E82, healthcare professional, Huelva

For migrants who do not have a healthcare card, emergency care is the only way to access healthcare, and they will often wait until they have severe health problems before going, especially SMFs with irregular status.

Not getting a proper follow-up is the main problem caused by entering the system through emergency care, as the focus lies on solving immediate problems and symptoms, and follow-up is only done in severe cases. A healthcare worker from Lleida mentioned that if they know SMFs are only there for a short time, they will provide acute care and not get them a healthcare card or refer them to a specialist. Despite often entering through emergency care, participants also mentioned that many SMF patients are referred through primary care, have insurance, and use the system correctly.

#### 3.2.2 Entering healthcare with other people´s documents

Participants mentioned that the difficulties in obtaining healthcare cards lead some people to try to access healthcare services by using other people's healthcare cards.

*“I simply inform my superiors or the hospital that it's not their card and give one piece of advice: "Man, this is a crime. Do you understand? For example, if you are sick and the person who gave you the card is diabetic or has sugar or allergies, do you understand? Or if you have an allergy, the doctor will prescribe you a medicine that will damage you for the rest of your life.”* E32, Cultural mediator, Catalonia

### 3.3 Facilitators for accessing healthcare services

#### 3.3.1 Reducing administrative barriers to healthcare

##### 3.3.1.1 Reinforcing universal access

Regardless of their legal status, all people in Spain are entitled to emergency care. In addition, participants from Catalonia said that SMFs received all the medical care they need, even without having a healthcare card.

“They do receive everything that is needed. It depends on the severity and what they ask for, but both those who have a regular administrative situation and those who don't receive care equally. They get everything they need, really.” E54, cultural mediator, Lleida

##### 3.3.1.2 Simplifying the process of obtaining healthcare cards

Facilitators were issuing healthcare cards for everyone with an identity card (in Andalusia) and coordinating between agricultural enterprises and the healthcare system to provide healthcare cards. La Rioja simplified the process of getting a displaced person healthcare card.

“*There are seasonal workers who are totally regulated. They come from other autonomous regions, and there is no problem because they are like displaced persons. Coming from another autonomous region, you have your [healthcare] card, a record is made for you, you are given a history of a displaced person, and you have all the assistance you need.*” E53, social health worker, La Rioja

##### 3.3.1.3 Permanent health card after obtaining permanent residence

When providing proof of having lived in Spain for at least three years and showing a working contract, migrants can apply for a residence permit (*arraigo social)*, which entitles them to a healthcare card that does not expire as long as they have a municipality registration.

“*If you have a residence permit, you are already paying social security contributions, and you are entitled to a healthcare card that does not expire.*” – E6, cultural mediator, Almería

#### 3.3.2 Patient centered care

##### 3.3.2.1 Willingness of healthcare service personnel to attend to migrants

Healthcare workers were willing to attend to SMFs, also when undocumented, according to the participants. In Catalonia, a healthcare worker mentioned that if SMFs did not have a healthcare card, they would be treated regardless. If needed, they would put a fictitious address in the system, for example, for a referral to specialist care. Participants from La Rioja and Catalonia mentioned never having seen a case where SMFs were not attended.

“*I don't know of any cases, but in other autonomous regions, some doctors are moral objectors [to applying the law], meaning they will attend a person if they come to them.*” – E22, cultural mediator, La Rioja

An employee of a hospital in Andalusia with a large migrant population said that there was much effort in integrating SMFs and improving access to healthcare.

##### 3.3.2.2 Attendance without appointments

Another facilitator was flexibility with appointments. A healthcare worker in Andalusia explained that they were very flexible in attending to SMFs without appointments.

“*We have a very flexible practice and try to see all patients when they come in for a walk-in consultation. So, perhaps 70 or 80 percent of the patients we see daily have scheduled appointments. However, a percentage of unexpected, temporary patients return from the campaign and show up at the door.”* - E4, healthcare professional, Andalusia

#### 3.3.3 Support services by NGOs

##### 3.3.3.1 Support obtaining healthcare cards

In Andalusia, several NGOs and associations supported migrants in obtaining a healthcare card. If a municipality registration was requested, some NGOs registered people or created identity documents for migrants without a passport, so the healthcare center could provide them with healthcare cards.

“*They [the NGO] have an agreement with the health center. I understand they have their requirement to be able to [access], but the person who really lives there, they usually give them a card that says they have three months and so on.*” - E37, cultural mediator, Andalusia

In Catalonia, several organizations, including the local health authorities and NGOs were working together to provide healthcare cards for SMFs even without municipality registration.

##### 3.3.3.2 Support with healthcare visits

In all regions, NGOs had professionals or volunteers to accompany migrants to the healthcare center and mediate between migrants and professionals at the health centers. A social worker pointed out that transactions at the hospital are a lot easier when supported by a third party. As NGOs were present in the shanty towns, they could identify SMF having problems with accessing healthcare and contact the healthcare center and mediate.

“*For the people we find in the settlements or in the programs of integral care for immigrants who have difficulties in accessing health care, if we think that this difficulty can be overcome because it is an error or because they have the right to demand access, we make a mediation by telephone, calling the health center and contacting the person with whom they had the problem*.” E73, NGO, Murcia

##### 3.3.3.3 Logistical and financial support

Some NGOs also provided transport to healthcare facilities if needed, although this was often arranged within their community. Participants from Andalusia and Murcia mentioned that NGOs provided support obtaining and paying for medication.

“*We call them [the healthcare service] if their back hurts and they need a pill, we do the mediation for them, and they have their medicine on their card and can buy it.*” - E65, cultural mediator, Almería

##### 3.3.3.4 Collaboration between healthcare centers and NGOs

Participants explained how collaborations between NGOs and healthcare facilities improved the effectiveness of healthcare. Some centers organized working groups, including several NGOs. In addition, an administrative worker at a healthcare center shared that they had a group chat with NGO workers and organized meetings on relevant topics; for example, when procedures changed, they followed training together to better educate and support migrant populations. Another good practice was NGOs supporting a healthcare center in following migrants' medication use by calling or visiting patients and answering their questions. A healthcare worker emphasized that they benefitted significantly from the collaboration with NGOs, helping SMFs fulfill their basic needs with health promotion programs and supporting them in contacting healthcare services.

“*We organize a meeting and bring together all the NGOs in the area, the City Council's Social Services, and ourselves. Then, we touch on all the points.*” E47, administrative healthcare worker, Almería

#### 3.3.4 Professionals’ suggestions for improvement of healthcare for SMFs

Several cultural mediators stated that a change in regulations was needed to solve the problems of obtaining a healthcare card for SMFs. As SMFs moved around and often required a registered address or a house, they suggested giving them a document stating they are seasonal workers entitled to healthcare. Another proposed solution was to allow people to register at the shanty towns.

From the healthcare system perspective, a system could be created to homogenize patient records from different autonomous communities, as healthcare workers did not have access to patients' medical histories from other autonomous regions.

“*This is a big problem, as patients' clinical history depends on autonomous regions. There were discussions of creating a single clinical history [but it has not yet been carried out]. -* E53, Social health worker, La Rioja

## 4 Discussion

The main barrier mentioned by professionals regarding access to healthcare in this study was the requirement to show a municipality registration, especially when living in informal settlements or moving between regions. Facilitators to overcome these barriers included simplifying administrative processes and support from NGOs in getting healthcare cards and residence permits. Other barriers were related to difficulties with reaching healthcare services, including lack of transport to health care facilities, centers being only open at SMFs’ working hours, financial barriers high out-of-pocket expenses when buying medications, and lack of time for healthcare staff to adequately explain processes within the healthcare system to SMFs. Facilitators included flexibility with appointments and willingness by healthcare personal to support migrants as well as logistical and financial support by NGOs.

Problems with municipality registrations and renting have been widely reported by migrants across Spain. For example, in a previous study women living in shanty towns in Andalusia reported not being allowed to register in their municipalities (Plaza Del Pino et al., 2023). Additionally, problems were faced getting a municipality registration living in informal housing or when not possessing an identity document and (Fundación Cepaim, 2023; Hsia and Gil-González, 2021). Furthermore, as mentioned in our study, discrimination in the housing market has been reported across Spain (Plaza Del Pino et al., 2023). While these barriers are experienced by all types of migrants, SMFs are especially affected by them as they migrate for work and potentially face them every time they move to another autonomous region.

This study found that while certain autonomous regions, such as Andalusia, facilitated the process of obtaining a healthcare card, others, including La Rioja, improved access to healthcare services for displaced SMF from other regions of Spain by allowing healthcare transfer without requiring a residence permit. The Spanish government issued several recommendations and regulations aiming at facilitating the municipality registration process, however, findings from this study indicate these are not always respected by municipal authorities. In 2019, the Ministry of Health recommended that health authorities should accept alternative documents as proof of residence for migrants without a municipal registration in Spain (e.g., travel letters issued by the consulate, school registrations, visits to social services) (Asesoría y Tutela Juridica a Migrantes - Legislación 2023). However, findings from this study indicate these are not always respected by municipal authorities. With the exception of most facilities in Andalusia, most healthcare centers from other regions continued to require residence permits, although there was considerable variation between centers within autonomous regions. Likewise, the Resolution of the Ministry of the Presidency of April 2020, allows registration in substandard housing, such as shacks and caravans (Agencia Estatal Boletín Oficial del Estado 2023), meaning that informal housing should not inhibit from getting a municipal registration, which was often not respected in all included autonomous regions. Furthermore, the requirements for obtaining a healthcare card, such as proving three months of residence, were interpreted differently by public officials throughout Spain leading to inconsistent implementation (Hsia and Gil-González, 2021). Compliance with recommendations and regulations from the Spanish government as well as consistency in the application of these regulations would reduce barriers to healthcare access for SMFs.

Besides healthcare system factors, SMF´s social determinants of health affect their access to healthcare services. Precarious working conditions, poor job security and high economic needs have been reported among SMF throughout Europe (HN Urrego-Parra et al., 2022). As a result, migrants might not want to take time off work because of the opportunity for (additional) income or fear of making a bad impression on their employer (Navarro-Gambín and Jansen, 2023). Fear of unemployment can cause presenteeism, working while sick, and discourage health-seeking behaviors (Galon et al., 2014; Gil-Gonzalez et al., 2015). Moreover, workplaces being far away from healthcare centers (Jiménez Lasserrotte M del and Ruiz Fernández, 2022) and varying working hours made it hard to schedule healthcare visits (Navarro-Gambín and Jansen, 2023). As our results show, these barriers can be partially overcome by being more flexible with rescheduling appointments and attending to SMFs without appointments. However, this is challenging considering the limited capacity of healthcare centers and the high burden for healthcare workers.

Several factors that limit access to healthcare simultaneously prevent migrants from overcoming their precarious labor conditions. These include a lack of cultural capital, including a low level of the Spanish language and low or unrecognized professional qualifications (Navarro-Gambín and Jansen, 2023). Occupational precariousness is socially reproduced as jobs in the informal labor market traditionally do not allow migrant workers to acquire legal status (Rousseau et al., 2008). Moreover, their irregular status prevents them from improving their labor conditions (López-Jacob et al., 2010). Undocumented migrants lack welfare support, which has been associated with decreased odds of general healthcare service use (Juárez et al., 2019). In Spain, migrants with an irregular status make less use of healthcare services compared to documented migrants, as shown in a study from Aragon (Gimeno-Feliu et al., 2021), or compared to the native-born population, as shown in a study from Catalonia (Dalmau-Bueno et al., 2021). Thus, irregular status is an additional barrier to good health and access to healthcare services.

In our study, facilitators and positive perceptions about the healthcare access of SMFs in Spain also emerged, with several professionals praising the overall healthcare provision to migrants. A study in shanty towns in Andalusia found that 83 % of the population knew their right to healthcare, and >78 % had a healthcare card (Navarro-Gambín and Jansen, 2023). This could be explained by Andalusia not requiring a municipality registration for obtaining the healthcare card and NGOs' work conducted in these areas, which was one of the main facilitators for access to healthcare identified in our study. A review on the role of institutional and non-institutional organizations in migrant health in the European WHO region found that while governmental organizations were mainly involved in research and policy-making, NGOs were active in providing healthcare and mediating between healthcare institutions and migrants (Ingrosso et al., 2015). This bridging role by NGOs in supporting migrants when they have issues accessing governmental services (e.g., with municipality registration) and healthcare services has proven to be essential. Other factors that can improve access to healthcare services for labor migrants include personal and work-related factors such as regularization, higher socioeconomic status, health insurance, labor union membership, and safe working conditions (World Health Organzation 2015). Finally, participants in this study emphasized the importance of sharing patient records between regions. Interoperability of the national healthcare system is one of the priorities of the digital health strategy of the Spanish national health system (Ministerio de Sanidad 2025) and progress is being made with the development of a virtual healthcare card, digital clinical records and electronical prescriptions that facilitate data sharing between the different autonomous communities (Ministerio de Sanidad 2024). However, there is currently a lack of a centralized interoperable platform what slows down the potential coordination for data sharing of critical health information. Each autonomous region uses different software and levels of digital maturity and resources vary, which lead to a lack of interoperability. The lack of a legal framework for health data governances and issues regarding privacy further exacerbate challenges around data sharing (EIT Health 2023; ICGL 2024).

### 4.1 Strengths and limitations

The main strength of this study was the high number of interviews (*n* = 92), including professionals from different areas and professions and therefore including a broad spectrum of views. However, the study also had some limitations. First, interviews were conducted during the COVID-19 pandemic, so some of the observations about SMFs' healthcare access may have been influenced by the general health situation at that time. Nevertheless, the interview questions focused on participants were also asked retrospectively about the pre-pandemic period, aiming to understand the situation understood as normality. Study findings related to the COVID-19 pandemic have been published elsewhere (Úbeda et al., 2025). Second, barriers and facilitators are only described from the professionals' point of view, not the perspectives of SMFs. The next phase of the AGROMISALUD project (Briones-Vozmediano et al., 2022) will explore the voices and experiences of the migrants themselves. Finally, this study does not explore differences regarding the country of origin or gender, which may be interesting since some migrant groups may be more vulnerable to experience inadequate healthcare provision. These issues did not emerge during the interviews in the current study but both aspects are also being specifically considered in the next phase of the AGROMISALUD project.

## 5 Conclusions

Despite being available for SMFs, services are not always accessible due to several identified barriers. The first step towards increasing access should be consistent compliance by public officials throughout all regions with the Spanish government’s recommendations and regulations regarding municipality registration. Furthermore, a more unified approach regarding SMFs' access to healthcare is needed between regions, including flexibilization of administrative processes and data sharing. Support services by NGOs and collaboration between NGOs and healthcare services improve access to healthcare, and this should be expanded to other regions. For optimizing access to healthcare for SMFs, the involvement and commitment of multiple stakeholders are required, including law and policymakers, employers, labor organizations, and health systems.

## Funding

This study has been funded by Instituto de Salud Carlos III (ISCIII) through the project PI20/01,310 (Co-funded by European Regional Development Fund/European Social Fund “A way to make Europe”/“Investing in your future”).

## CRediT authorship contribution statement

**Lena van Selm:** Conceptualization, Writing – original draft, Writing – review & editing, Formal analysis. **Iratxe Pérez-Urdiales:** Conceptualization, Formal analysis, Investigation, Writing – review & editing. **José Tomás Mateos:** Conceptualization, Writing – review & editing, Investigation. **Maria del Mar Jiménez-Lasserrotte:** Writing – review & editing, Conceptualization, Investigation. **María del Mar Pastor-Bravo:** Investigation, Conceptualization, Writing – review & editing. **Ana Requena-Méndez:** Writing – review & editing, Conceptualization. **Erica Briones-Vozmediano:** Writing – review & editing, Investigation, Conceptualization, Supervision, Writing – original draft.

## Declaration of competing interest

The authors declare that they have no known competing financial interests or personal relationships that could have appeared to influence the work reported in this paper.

## Appendix Supplementary materials

Image, application 1
