# Supplementary figures and images for: Accessibility of healthcare services for seasonal migrant farmworkers in Spain: barriers and facilitators identified by professionals
Source: J Migr Health. 2025 Aug 18;12:100353. doi: 10.1016/j.jmh.2025.100353 (PMC12747175; doi:10.1016/j.jmh.2025.100353)

**Supplementary figure 1: mentions of experts by sub-theme**


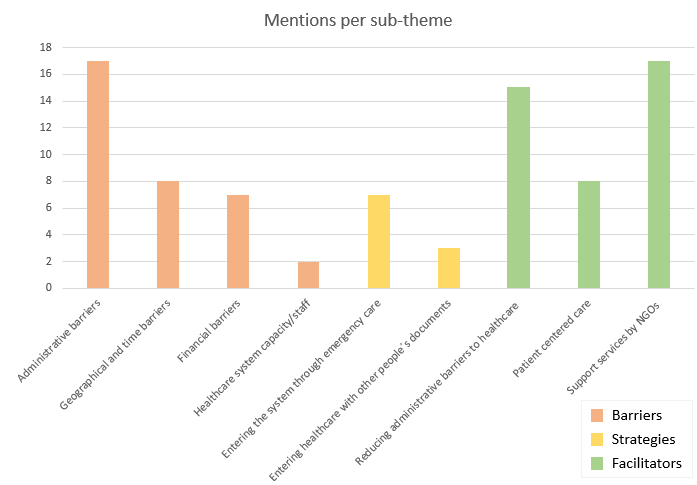

Supplement: Supplementary file 1 [file mmc1.docx]
